# Wireless agents for brain recording and stimulation modalities

**DOI:** 10.1186/s42234-023-00122-5

**Published:** 2023-09-20

**Authors:** Ilhan Bok, Adam Vareberg, Yash Gokhale, Suyash Bhatt, Emily Masterson, Jack Phillips, Tianxiang Zhu, Xiaoxuan Ren, Aviad Hai

**Affiliations:** 1 Department of Biomedical Engineering, University of WI – Madison, 1550 Engineering Dr, Madison, WI Rm 2112 USA; 2 Department of Electrical and Computer Engineering, University of WI – Madison, Madison, WI USA; 3 Wisconsin Institute for Translational Neuroengineering (WITNe), Madison, WI USA

**Keywords:** Microscale, Nanoscale, Electromagnetic, Magnetoelectric, Injectable, Implantable, Nanoparticles, Neuroimaging, Radio frequency (RF), Magnetic resonance imaging (MRI), Ultrasound imaging

## Abstract

New sensors and modulators that interact wirelessly with medical modalities unlock uncharted avenues for in situ brain recording and stimulation. Ongoing miniaturization, material refinement, and sensitization to specific neurophysiological and neurochemical processes are spurring new capabilities that begin to transcend the constraints of traditional bulky and invasive wired probes. Here we survey current state-of-the-art agents across diverse realms of operation and evaluate possibilities depending on size, delivery, specificity and spatiotemporal resolution. We begin by describing implantable and injectable micro- and nano-scale electronic devices operating at or below the radio frequency (RF) regime with simple near field transmission, and continue with more sophisticated devices, nanoparticles and biochemical molecular conjugates acting as dynamic contrast agents in magnetic resonance imaging (MRI), ultrasound (US) transduction and other functional tomographic modalities. We assess the ability of some of these technologies to deliver stimulation and neuromodulation with emerging probes and materials that provide minimally invasive magnetic, electrical, thermal and optogenetic stimulation. These methodologies are transforming the repertoire of readily available technologies paired with compatible imaging systems and hold promise toward broadening the expanse of neurological and neuroscientific diagnostics and therapeutics.

## Background

Recent progress in neural engineering has narrowed the gap between invasive precision neurotechnologies and noninvasive methods for imaging and stimulating the brain. Robust brain machine interfaces (BMIs) primarily rely on mainstay electrophysiological tools including microelectrode arrays (Chaudhary et al. 2022; Spira and Hai 2020; Willett et al. 2021), electrocorticography (ECoG) arrays (Merk et al. 2022) and other implantable shanks (Paulk et al. 2022) that often elicit a cascade of immunological responses resulting in cellular disruption and damage to the brain parenchyma (Shen et al. 2023). Despite pioneering high density designs (Steinmetz et al. 2021), wired approaches currently preclude the combination of volumetric coverage of bulk nervous tissue with intrinsic sub-microscale spatiotemporal capabilities. Furthermore, while tethered options for therapeutic deep brain stimulation represent significant medical solutions for severe brain conditions such as Parkinson’s disease and epilepsy (Krauss et al. 2021), they also require invasive surgical procedures and periodic disruptive battery changes. In contrast, noninvasive neuroimaging and modulation modalities such as functional magnetic resonance imaging (fMRI), transcranial magnetic stimulation (TMS), focused ultrasound (fUS), magnetoencephalography (MEG), functional near-infrared spectroscopy (fNIRS) and others (Talebloo et al. 2020) can deliver broad coverage while eliciting minimal damage to the nervous system. However, they have long suffered from low specificity or impeded spatiotemporal resolution ill-suited for providing direct access to the inner workings of the brain. Recent advances in wireless device architecture (Khalifa et al. 2021; Won et al. 2021) and molecular imaging agents (Rabut et al. 2020; Wei et al. 2021) present approaches that merge the volumetric encoding capabilities of noninvasive modalities with the electrophysiological and molecular specificity of micro- and nano-scale probes and are beginning to bridge this technological gap.

**Fig. 1:**
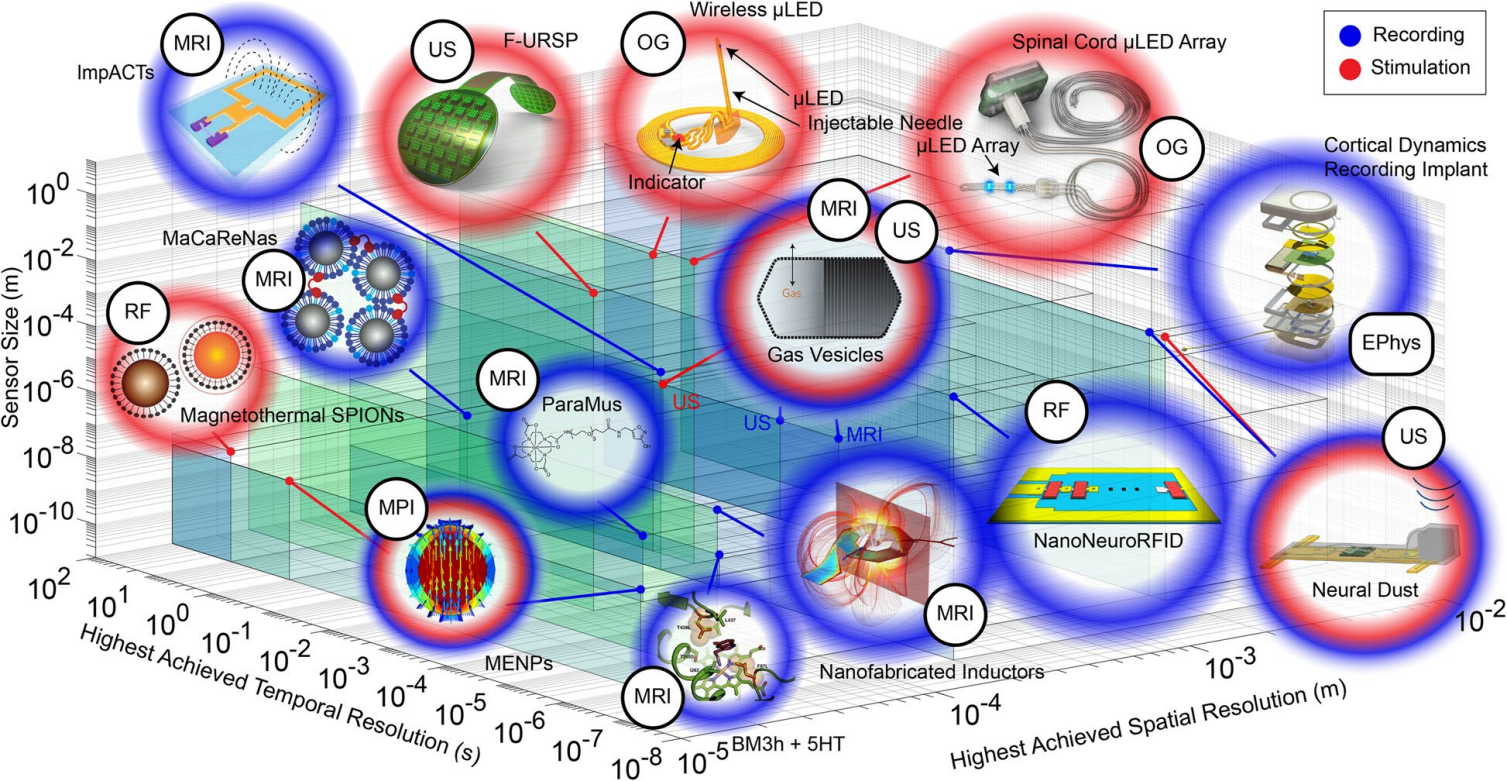
Overview of the approximate sizes and spatiotemporal resolutions of neural transducers. Blue halos indicate probes used for recording, red halos indicate stimulation and bicolor halos indicate dual recording/stimulation capability, smaller black circles indicate corresponding imaging techniques (MRI = magnetic resonance imaging, MPI = magnetic particle imaging, US = ultrasound, EPhys = electrophysiology, RF = radiofrequency, OG = optogenetics). Tinted blue-green planes are included to facilitate context determination of spatial resolution (x-axis), temporal resolution (y-axis), and sensor size (z-axis). See the corresponding data in Table 1 for precise values

Improvements in tetherless probe design revolve around the minimization of size and invasiveness for delivery, the optimization of signal transfer and power deposition in tissue, and the maximization of sensitivity, specificity, spatiotemporal resolution and hermiticity (Shen et al. 2023; Vázquez-Guardado et al. 2020). These refinements are achieved through exploration of micro- and nano-synthesis techniques spanning new strategies in electronic engineering, material composition, particle synthesis and rational molecular design. In this review, we highlight recent state-of-the-art wireless neural recording and stimulation agents, focusing on modality-specific operation and features. For probes relevant to each readout method, stimulation technique or both (Fig. 1), we specify the geometric attributes, composition, *modus operandi*, and spatiotemporal resolution. We conclude with opportunities for cross-modality integration towards a comprehensive tool set in neuroscience.

**Table 1:** Summary of the imaging technique(s), size, and spatiotemporal resolution of neural transducers

Probe	Recording/Stimulation Technique	Size (m)	Achieved Spatial resolution (m)	Achieved Temporal Resolution (s)	Stim/Record (S/R)
Neural Dust	US (Seo et al. 2016)	1.00E-03	2.00E-03	1.00E-06	R
StimDust	US (Piech et al. 2020)	1.00E-03	2.00E-03	5.40E-07	S
Gas Vesicles	MRI (Lakshmanan et al. 2017)	1.00E-08	1.00E-03	1.00E-02	R
Gas Vesicles	US (Shapiro et al. 2014)	1.00E-08	1.00E-03	1.00E-01	R
Gas Vesicles	US (Hou et al. 2021)	1.00E-08	1.00E-03	1.00E + 01	S
ImpACTs	MRI (Hai et al. 2019)	1.00E-05	2.00E-04	1.00E-02	R
MaCaReNas	MRI (Okada et al. 2018)	1.00E-07	1.00E-04	1	R
ParaMus	MRI (Bricault et al. 2020)	1.00E-09	1.00E-04	1.00E-03	R
BM3h	MRI (Hai et al. 2016; Lee et al. 2014)	1.00E-10	2.00E-04	1.00E-03	R
Nanofabricated Inductors	MRI (Phillips et al. 2022)	1.00E-06	**4.00E-05**	**1.00E-06**	R
Magnetoelectric Nanoparticles	MRI (Kaushik et al. 2019), MPI (Bok et al. 2022; Guduru et al. 2018)	1.00E-08	**2.00E-05**	**1.00E-06**	R
Magnetoelectric Nanoparticles	RF (E. Zhang et al. 2022a, b)	1.00E-08	**2.00E-05**	1.00E + 00	S
NanoNeuroRFID	Magnetoelectric (Zaeimbashi et al. 2019)	1.00E-03	2.00E-04	1.00E-07	R
Cortical Dynamics Recording Implant	Electrophysiology (Borton et al. 2013)	5.60E-02	1.00E-03	1.28E-04	R
Magnetothermal SPIONs	RF (Chen et al. 2015)	2.20E-08	**2.00E-05**	1.00E + 01	S
F-URSP	US (Jiang et al. 2022)	4.40E-02	6.40E-05	1.00E-03	S
Wireless µLED	OG (Shin et al. 2017)	1.00E-02	2.70E-04	5.00E-02	S
Spinal Cord µLED Array	OG (Kathe et al. 2022)	1.50E-02	2.70E-04	1.00E-02	S

Values in **bold** are theoretical bounds but have not yet been achieved. They are included for comparison purposes

## Wireless micro- and nano-electronic devices

Electronic recording and stimulation devices have unlocked outstanding capabilities in neuroscience and neurology culminating in clinical BMIs capable of single-neuron level interfacing for handwriting prediction (Willett et al. 2021) character selection-based mind-controlled typing (Chaudhary et al. 2022) and sensation-based control of robotic prosthetic limbs (Flesher et al. 2021). These platforms have recently implemented high density neuropixel probes for closed loop single unit recording and stimulation in humans (Paulk et al. 2022; Topalovic et al. 2023) and are now increasingly integrated with wireless transmission circuitry and on-board battery power. Even though these innovations eliminate most extracranial wiring and facilitate free patient movement, their overall footprint and invasiveness remain considerably sizeable. To mitigate these limitations, new wireless architectures that focus on inductive power delivery to single motes are enabling completely encapsulated devices (< 0.2 mm^3^) that can be delivered by injection (Khalifa et al. 2021). Recent work demonstrates individually addressable integrated circuit (IC) microchip modules with significantly smaller dimensions (650 μm × 650 μm × 250 μm, or 0.1 mm^3^) termed “neurograins” that are powered at the RF regime via a 1 GHz transcutaneous communication relay coil and capable of bidirectional recording and stimulation (Jihun Lee et al. 2021a, b). With predicted scalability up to a swarm of 770 individual sensor units and high sensitivity (noise floor 2.2 µV_rms_, sampling rate 1 kHz), epicortical recordings were demonstrated in rat motor and sensory cortices in conjunction with intracortical stimulation of nearby regions. The devices were deposited onto the cortex manually following craniotomy, but with optimized efficiency at the midfield range relying on both inductive and radiative modes for considerably smaller devices (Ho et al. 2014), this approach can potentially allow for delivery by injection and larger device population assuming high resolution (sub-micron) complementary metal-oxide semiconductor (CMOS) processes is used for future fabrication.

Similar uplink approaches were shown for radically different readout modalities, most recently for fUS-mediated recording and stimulation using implanted motes (Piech et al. 2020; T. Zhang et al. 2022a, b). Taking advantage of micrometer-scale ultrasonic wavelengths, energy harvesting and signal transfer can be facilitated by piezoelectric transducers implanted in nerve tissue with relatively small device footprint (750 μm × 750 μm × 750 μm, or ~ 0.4 mm^3^) demonstrating electroneurography and electromyography with sufficient sensitivity (noise floor of 180 µV_rms_, adjustable pulse repetition frequency from 0 to 5 kHz). Further improvements to material composition were shown for optimized signal transfer in tissue empowering fUS-based wireless devices for deep brain stimulation (T. Zhang et al. 2022a, b). By sandwiching a 6 × 6 array of samarium-doped crystals coated with gold/chromium between a mesh of stretchable copper electrodes and biocompatible polydimethylsiloxane (PDMS) barriers, highly efficient ultrasonographic power transfer was shown for stimulation-driven analgesia counteracting formalin-induced pain up to 10 days in vivo (stimulation temporal resolution ranging from 0 to 120 Hz). Further such strategies for maximizing US power transfer and increasing long term biocompatibility can better leverage high ultrasonic signal propagation at tissue-compatible wavelengths. Other strategies for maximizing wireless power can leverage enhanced coupling between the device and biological substrates and more efficient transmission circuitry and pulse sequences. Peripheral nervous system applications for fUS are particularly unhindered by tissue power deposition constraints and therefore more conducive to potential clinical use, as is exemplified in piezoelectrically sensitive elements serving as a superior wireless retinal prosthetic architecture (Jiang et al. 2022). The system presents a flexible array with considerably higher spatial capabilities compared with existing devices (up to ~ 0.1–1 mm @ 1–10 MHz) and a maximal signal-to-noise ratio of 32.6 dB, holding promise for transducing fUS input pulses to electrical signals as a new method for videographic-based vision restoration.

Additional unique designs of standalone wireless electronic circuits for other modalities were shown recently for modulating MRI contrast in response to brain activity, leveraging deep tissue three-dimensional encoding capabilities of the modality through bone without requiring transcutaneous uplink mediator coils (Bhatt et al. 2023; Hai et al. 2019). By coupling responsive field effect transistors (FETs) such as ion-sensitive FET (ISFET) with wireless circuits resonating at MRI resonance frequencies (< 1 GHz), implantation and readouts (145 Hz sampling rate) within rat somatosensory cortex were demonstrated in vivo by detecting tuning and Q factor alterations that coincide with changes in extracellular ionic concentration in the cortex during peripheral stimulation (Bhatt et al. 2023). This approach is compatible with either simple near field antennas or more sophisticated MRI RF hardware for 3D encoding of multiple devices at clinical magnetic fields, yielding detectability with lower bounds of 7.57 nM. Future optimization of resonator and FET gate geometry for on-chip integration, material formulation and chemical functionalization, will allow for further customization of tuning sensitivity, resolution, and specifically-targeted analytes (Jasanoff et al. 2020).

Achieving cross-modality brain sensing and modulation on the same miniaturized wireless device has been a challenging endeavor for electronic circuit design due to component complexity and number of elements impacting overall size and invasiveness. Promising optoelectronic approaches involving the genetically customizable toolkit of optogenetics (Boyden et al. 2005; Pavlov and Tracey 2022) that could in principle be integrated on a single chip exemplify a wireless, battery-free implantable device for multimodal stimulation and electrochemical sensing (Stuart et al. 2023). Using specialized flexible implants considerably smaller than previous examples but still sizeable at 12 mm × 8.5 mm × 3.2 mm, devices containing both onboard µLED for optogenetic stimulation and Nafion-coated carbon nanotube electrodes for detecting dopamine (sampling rate 25 Hz) were implanted into murine striatum and used to perform optogenetic stimulation in parallel with transient dopamine measurements. With high sensitivity to concentrations as low as 1264.1 nA μM^−1^ cm^−2^ DA, dopamine increase, partial reduction, and stabilization in response to morphine injection and subsequent reversion to baseline upon injection of naloxone were demonstrated in freely moving animals. Another recent study incorporating electromyography, electroencephalography, optogenetics, thermal sensing, and microfluidic drug delivery into a single implantable module 40 mm × 18 mm × 3 mm large allows for closed-loop control of activity with impressively low noise levels of 3.0 μV_PP_ at 256 Hz (Ouyang et al. 2023). This module was used in rats for in vivo recording of neural activity during sleep and to automatically release midazolam to temporarily suppress pilocarpine-induced seizures. This adds to increasingly more capable designs for multi-modal functionality on the same wireless device that could provide complex read–write agents for tapping into the brain with reduced invasiveness.

Further miniaturization of wireless micro- and nano-scale devices for accessing the nervous system will depend on new geometries at finer feature resolution relaying signals at the cellular-scale with minimal power requirements. A recent example proposes passive nanofabricated spiral coils interfaced with single neurons as magnetic transducers potentially opening the door to MEG-mediated *in-situ* sensing of neural activity at unprecedented spatiotemporal resolutions (Phillips et al. 2022). Other creative geometries can also augment signal transfer as demonstrated by fractal patterns fabricated on stretchable and flexible electronics (Luo et al. 2023). Applying optimized component geometries and highly tailored device topology for tissue power harvesting in neural implants (Xu et al. 2022) can also minimize device crosstalk and maximize resolution for applications such wireless retinal stimulators nearing natural visual resolution (Wang et al. 2022). Additionally, flexible and stretchable substrates present a bioresorbable option for peripheral nerve modulation and near field wireless electroencephalography that could be applied for CNS applications (Song et al. 2023).

Electronic devices for neurotechnology present order 10^1^ – 10^3^ Hz temporal resolution and unmatched capabilities as platforms for signal transfer and conversion in the brain, but are greatly limited in delivery, clear-out, and precise penetration to relevant submicron structures. In order to target the brain parenchyma and subcellular somatic and synaptic space for neurotransmitter detection and single cell interfacing, responsive nanoscale particles can yield far greater neuroanatomical coverage and enable mass production of large batches of agents without necessitating highly involved fabrication and device upkeep procedures. We outline these aspects regarding specific nanoparticle sensor schemes in the following section.

## Nanoparticles as wireless dynamic contrast agents

Nanoparticle (NP)-based agents are readily delivered by injection, and depending on their chemical properties, can cross the blood–brain barrier (BBB) into target anatomical and intrasynaptic regions in the brain either naturally, through functionalization and endocytosis, or by applying osmotic, electromagnetic or ultrasonic field gradients (Hersh et al. 2022). The absence of complex IC thin-film electronic components lends NPs with a significantly less invasive, isotropic geometry that insinuates spatially and rotationally homogeneous fields simplifying readout interpretation, signal relay and multi-modality biological validation. In addition, NPs, nanostructures, and nanotransducers can be used as sensors and stimulators in tandem with or even integrated into electronic devices.

The use of NPs as standalone dynamic contrast agents in the brain evolved initially for MRI-coupled sensing by particle aggregation mechanisms, yielding an eclectic toolkit of semisynthetic dynamic sensors demonstrated for detecting diverse analytes (Wei et al. 2021). Deep brain calcium readouts mediated by spherical superparamagnetic iron oxide nanoparticles (SPIONs) functionalized with C2 domains of synaptotagmin were shown by way of reversible Ca^2+^ concentration-dependent NP aggregation resulting in local changes in MRI signal (Okada et al. 2018). This approach demonstrates calcium response following stimulation by local K^+^ or glutamate infusion, as well as direct striatal response during ipsilateral medial forebrain stimulation, and introduces a more clinically applicable method complimentary to ubiquitous optical calcium indicators. A similar approach was used for detecting neurotransmitters using NPs functionalized with heme-based proteins engineered via directed evolution (Hsieh et al. 2019). SPIONs conjugated to different protein variants with tailored affinity to specific monominergic neurotransmitters such as dopamine or serotonin, were shown to detach from their corresponding tyramine-based ligands in the presence of the neurotransmitter of choice and dynamically alter the MRI signal upon neurotransmitter release or reuptake. This work demonstrates a promising modular toolkit for MRI detection of neurochemicals relying on existing protein-based neurotransmitter sensors (Hai et al. 2016; Li and Jasanoff 2020) or other binding agents. Volumetric MRI readouts for this and similar techniques are achieved at sub-millimeter and second-scale spatiotemporal resolution (~ 4.6 s per map at 200 µm voxel size) and can be further improved towards the millisecond scale relevant to neuronal signaling using more sophisticated pulse sequences (Polimeni and Lewis 2021). A recent cross modality strategy exemplifies selective sensing of illumination in the brain by MRI detectable photosensitive liposomes relevant to photostimulation and phototherapy (Simon et al. 2023). By enclosing the contrast agent gadoteridol within liposomes with embedded light-sensitive 1-stearoyl-2-(4-(n-butyl)phenylazo-4’-phenylbutyroyl) phosphocholine (AzoPC), light photoisomerizes AzoPC agents between cis and trans states, allowing for modulation of permeability to water and consequently changing MRI T1 brain contrast readouts in vivo. This platform can be applicable broadly for detecting optical phenomena and injectable luminescent indicators across the brain without the requirement for invasive endoscopes or translucent tissue.

NPs have also proved to be a useful tool for delivering neuromodulation (Romero et al. 2022). Earlier seminal work established wireless magnetothermal deep brain stimulation (mDBS) in vivo by applying whole-brain alternating magnetic fields (*f* = 160 kHz) into locally injected SPIONs resulting in the activation of heat-sensitive capsaicin receptor TRPV1 channels and modulation of neuronal activity (Chen et al. 2015). This method has now been shown to enable alleviation of Parkinsonian-like symptoms by stimulating neurons in the subthalamic nucleus and reversing motor deficits in Parkinsonian mice models (Hescham et al. 2021). These studies delineate the efficacy of minimally invasive NP-mediated brain stimulation without the requirement for implanted hardware. Other neuromodulation approaches that take advantage of mechanosensitive ion channels apply magnetic torque onto spherical polysterene nanospheres embedded in octahedral SPIONs (m-Torquers, *d* =  ~ 500 nm, τ_B_ = 0.032 s or 31.25 Hz) for remote brain mechanostimulation (Jung-uk Lee et al. 2021a, b). Actuation of brain injected m-Torquers in the motor cortex of freely moving mice is achieved by applying a rotating pulse in a spatially inhomogeneous field configuration resulting in reversible calcium activation. Additional cross-modality approaches provide precise spatiotemporal control of optogenetic brain stimulation using injectable ultrasonographically actuated fluorescent ZnS nanoparticles that are charged wirelessly by light emitting diodes and only fluoresce under fUS pulses here applied at 1 Hz with 0.7 mm × 0.7 mm in-plane focus (Wu et al. 2019).

Chemically functionalized static NPs offer new avenues for specificity and biologically stable wireless recording and stimulation, but present safety limitations related to specific absorption rate (SAR) and excess heat dissipation (Tong et al. 2017). A new class of dynamic interfacially-coupled magnetoelectric heterostructures are emerging as simple and efficient actuator components possessing unique inherent capabilities for electromagnetic field detection and delivery of power for stimulation, thereby raising spatiotemporal resolution while simplifying sensor assembly. In the following section we touch upon the utility of magnetoelectric materials for brain technologies.

## Magnetoelectric sensors and stimulators

Emerging magnetoelectric materials can be efficiently modulated by remote electric or magnetic alternating fields in the presence of a bias magnetic field, with rapid responsiveness at picosecond timescales, heralding new capabilities in diverse fields including bioelectronic medicine (Yue et al. 2012; Hadjikhani et al. 2017; Singer et al. 2020; E. Zhang et al. 2022a, b). Injectable bio-compatible magnetoelectric nanoparticles (MENPs) comprising a magnetostrictive CoFe_2_O_4_ (CFO) core coated with a piezoelectric BaTiO_3_ (BTO) shell were specifically simulated (Yue et al. 2012) and shown in recent years (Kozielski et al. 2021; E. Zhang et al. 2022a, b) to facilitate wireless neural stimulation offering a possible alternative to current bulky clinical stimulators. MENPs were injected into the subthalamic region in mice for magnetic-field-controlled deep brain stimulation using relatively small fields (220 mT bias, 140 Hz 6 mT alternating) successfully eliciting local brain activity changes manifested behaviorally as alterations in gait and movement patterns (Kozielski et al. 2021). This work strongly suggests potential use for minimally invasive therapeutic amelioration of Parkinsonian symptoms. Additional demonstrations of synchronous neuronal firing activity driven by MENP stimulation at 20 Hz (E. Zhang et al. 2022a, b) present utilities for modulating neural activity at single neuron resolution. This study also demonstrates voltage sensitive dye measurements for detecting electric fields developing proximally to MENPs, opening avenues for opto-magnetoelectric imaging of neural electrical activity or magnetoelectrically-mediated optogenetic stimulation of the brain. MENPs were also recently proposed for brain recording as a specialized variant of magnetic particle imaging (MPI) also termed magnetoelectric particle imaging (MEPI) (Guduru et al. 2018). Quantifications of the MEPI neural readouts of injected CFO-BTO diffusing in brain tissue demonstrate the feasibility of structural and dynamic functional imaging of neural activity. Further analysis demonstrates optimized core–shell ratios informing future MENP synthesis that can enable direct volumetric imaging of electrophysiology with superior spatiotemporal resolution limited only by scanner performance (Bok et al. 2022). The ability of CFO-BTO MENPs to traverse the BBB and maintain relatively long term biocompatibility was affirmed using MRI-directed delivery in non-human primates at clinical fields (Kaushik et al. 2019). Entry into cerebral areas and deeper basal ganglia was confirmed as T2* signal decreases 3 h following intravenous injection. Moreover, blood profile toxicity verifications and work demonstrating minimal glial inflammatory activation following BBB penetration (Nguyen et al. 2021) point to the evolution of MENPs as a viable and broad clinical tool in the near future.

Magnetoelectric materials have also been used to augment wireless micro- and nano-electronic neural probes allowing for the measurement of very small magnetic fields in the brain. A hypersensitive thin bilayer consisting of ferromagnetic iron gallium boron (FeGaB) and piezoelectric aluminum nitride (AlN) were designed as a magnetoelectric microscale antenna sensor array loaded with complex onboard transmission and reception circuitry and theoretically able to record physiological fields with high sensitivity of up to 40 pT and 200 µm × 10 s to 100 s of MHz spatiotemporal resolution (Zaeimbashi et al. 2019). Further work affirm that the proposed highly miniaturized magnetoelectric antenna (250 × 174 µm^2^) has wireless power transfer efficiency between one and two orders of magnitude above existing miniaturized micro-coils and a lower limit of detection between 300 and 500 pT in ex vivo neural tissue (Zaeimbashi et al. 2021). These approaches can supersede standard antennas for neural implants replacing sensitive wired probes. This highly elevated power transfer was also shown to enable stimulation using magnetostrictive Metglas layer bonded to piezoelectric as a magnetically powered small (< 25 mm^2^) neural stimulators at 100–200 Hz (Singer et al. 2020). Stimulation of the subthalamic nucleus using such encapsulated wireless magnetoelectric bilayer implants modulated oxidopamine-induced Parkinsonian symptoms in freely moving rats. Furthermore, similar magnetoelectric-powered bio implants (ME-BITs) can be delivered non-surgically through a percutaneous catheter and enable frequency-dependent amplitude modulation-based endovascular stimulation at 1–10 Hz using strain-coupled metglas – lead zirconium titanate bilayer in rat sciatic nerve or pig femoral artery (Chen et al. 2022). The same proof-of-concept has been applied previously for peripheral neurostimulation up to 200 Hz mitigating neuropathic pain verified using GCaMP6s in vivo (Yu et al. 2020). Together, these approaches greatly simplify delivery, power relay and implementation of wireless circuitry.

## Dynamic microbubbles and gas vesicles

**Fig. 2:**
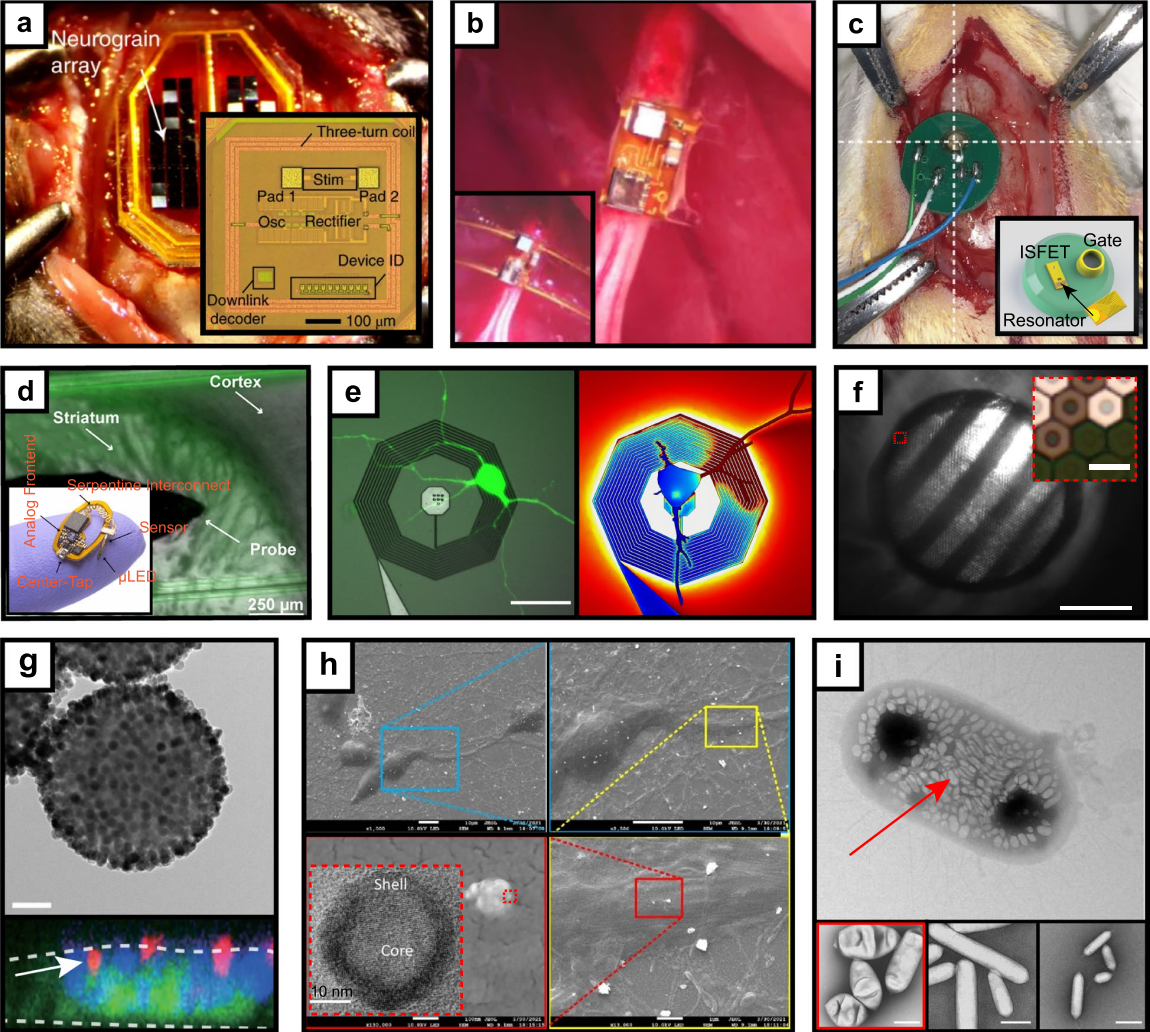
Recent wireless neural recording and stimulation agents. **a** Neurograins (Lee et al. 2021a, b), **b** Stim/neural dust (Piech et al. 2020), **c** Implantable active coil-based transducers (Bhatt & Masterson et al., 2023) **d** μ-LED and carbon nanotube for multimodal optogenetic stimulation and electrochemical sensing (Stuart et al. 2023), **e** Nanofabricated magnetic neuronal transducers (Phillips et al. 2022) (Scale bar: 30 µm). **f** Photovoltaic pixel array for retinal stimulation (Wang et al. 2022) (Scale bar: 1 mm, inset scale bar: 50 μm) **g** Transmission electron micrograph of m-Torquers (Scale bar: 100 nm) and c-Fos-tagged neurons showing presence of m-Torquers (Jung-uk Lee et al. 2021a, b) **h** Magnetoelectric nanoparticles for brain stimulation (Nguyen et al. 2021), **i** Responsive gas vesicles (GVs) as ultrasound reporters (Bourdeau et al., 2018), scale bars are 200 nm

An alternative to designs requiring exotic material formulations and involved fabrication processes, ultrasonography makes use of gaseous microbubbles and more recently biogenic gas vesicles (GVs) acting as fully biocompatible contrast agents for sensing and modulation at millisecond-scale precision (Rabut et al. 2020). Despite signal attenuation across the skull, intravenously injected microbubbles can provide high-resolution angiography and were recently shown to relay brain-wide functional hemodynamic activity by ultrasound localization microscopy (Heiles et al. 2022; Renaudin et al. 2022). Dynamic angiogenic readouts with considerably higher temporal and spatial resolution (~ 1 s, 6.5 µm, respectively) are enabled by averaging deconvolved transient sliding windows of US readouts followed by spatial registration, successfully extracting blood velocity, microbubble count and flux (Renaudin et al. 2022). Structural classification of these parameters into arterioles, venules, intra-parenchymal vessels, and pial vessels exhibited unique microbubble count, speed, and diameter profiles in the cortex that correlated with visual or whisker stimulation in the barrel cortex of rats. Further improvements of microbubble localization and corresponding brain readouts was achieved by optimized reconstruction algorithms establishing a method for sensitive imaging of cerebral vasculature and functional hemodynamic alterations in the brain (Heiles et al. 2022). Similarly to naturally formed microbubbles, GVs are nanometer-scale gas modules susceptible to fUS manipulation, have specialized shapes (Fig. 2i) that are receptive to conjugation for sensing diverse analytes, and can be genetically expressed for scalable synthesis (Maresca et al. 2018). Their impermeability to water allows for highly stable aggregation-based contrast enhancement across various modalities and implosion by applying sufficiently strong fUS energy for rapid disassembly and clearance(Lakshmanan et al. 2020). Some evidence of the use of GVs for deep brain stimulation comes in the form of low-intensity US-induced cavitation of injected GVs in ventral tegmental area of GCaMP6s mice, showing calcium responses following US application at 1 kHz (Hou et al. 2021). These experiments revealed changes in fluorescence not found from GVs without US or US with control saline injection alone, and added evidence of activation of mechanosensitive membrane channels as a possible mechanism of US neuronal stimulation at frequencies of up to 1 kHz (Yoo et al. 2022). Other promising avenues come in the form of enzymatically modulated GVs engineered to evoke US signals nonlinearly in response to proteases such the Ca^2+^-activated calpain (Lakshmanan et al. 2020) with high relevance to characterizing synaptic plasticity and neuropathologies such as Alzheimer’s disease. Magnetic field susceptibility artifacts surrounding GVs in water additionally allow for their use as contrast agents for MRI with repetition time on the order of seconds (Lu et al. 2018). Comparing GVs and control saline injections at opposing hemisphere in mice shows a reduction in T2* which vanishes when GVs are collapsed via acoustic pulses, while multiplexed acoustic modulation in MRI is feasible via co-injection of wild type GVs and GVs with outer scaffolding protein genetically removed, deepening the information content of neurologically-relevant readouts. Microbubbles and gas vesicles are useful not only as contrast agents but also as a strategy for opening up the BBB through cavitation, enabling perfusion of otherwise impermeable drugs and other active agents into brain parenchyma (Szablowski et al. 2019; Chen et al. 2021). MRI studies verify BBB opening 0.5 h after microbubble stimulation and transcranial fUS application that safely return to baseline after 24 h for 4 of 6 patients showing no adverse immunological responses (Chen et al. 2021). The ability of gas-based agents to function in dual modality scenarios, facilitate BBB delivery, and increasing evidence of their ability to report on diverse biophysical processes (Farhadi et al. 2021) holds further promise for interrogating neural tissue.

## Rational design of injectable molecular neural probes

Molecular units provide a particularly customizable and non-invasive pathway towards accessing neural dynamics across multiple modalities. Optical probes are powerful tools ubiquitous in research but challenging to implement clinically for deep brain regions and are beyond the scope of this review. Deep brain indicators for clinical modalities can be engineered through rational small molecule chemical synthesis or iterative directed evolution through protein mutagenesis and subsequent binding affinity optimizations (Wei et al. 2021). Early examples of specialized probes for brain neurotransmitter detection made use of mutated cytochrome P450-BM3 heme domain of genetically engineered bacteria for high affinity binding and sensitive MRI response to monoaminergic compounds (Shapiro et al. 2010). This family of protein sensors sprung variants such as BM3h-8C8 exhibiting physiologically relevant sensitivity (*r*_*2*_ of 4 mM^−1^ s^−1^) and easily detectable MR signal changes in response to exogenously-infused dopamine in the brain. This strategy later developed into a toolkit for molecularly specific functional visualization of endogenous neurotransmitters across large regions of the brain in vivo (Lee et al. 2014; Hai et al. 2016; Li and Jasanoff 2020). Fluctuations of dopamine in the nucleus accumbens of rats in response to electrical stimulation of the medial forebrain bundle specifically showed dopamine-dependent time-locked MR signal changes verified by amperometry, constituting the first molecular fMRI of neurotransmitter release (Lee et al. 2014). Corresponding experiments performed using a serotonin-binding variant yielded readouts of cellular reuptake across large striatal regions (Hai et al. 2016) allowing for real-time detection of selective serotonin and dopamine reuptake inhibitors as an promising tool for antidepressant drug discovery. These and similar responsive agents leverage the volumetric capabilities of MRI for sensing neurochemicals and provide detailed multi-slice maps of neurotransmitter dynamics in the brain that can reveal fundamentally new insights on brain circuitry and activity, and corresponding brain metabolism (Li and Jasanoff 2020). Other protein-based sensors showing promise for detecting ionic species in the brain employ competitive binding to naturally occurring proteins such as calprotectin, which sequesters Mn^2+^ ions when exposed to Ca^2+^ thereby generating calcium-dependent readouts (Ozbakir et al. 2021). Smaller peptide-based sensors come in the form of dopamine-sensitive biotinylated activatable vasoprobes consisting of a dopamine-binding immunoglobulin G opens further frontiers for sensing a diverse array of molecules and biological entities in vivo (Ohlendorf et al. 2020). Modulation using these sensing capabilities is possible by enabling switching of blocking domains sensitive to biophysical/biochemical events, allowing for actuation of genetic expression with fast temporal resolution, and inducing more rapid response of probe elements during modulation.

Rational design of small molecular probes for detecting and modulating neural signaling events generated several useful tetherless methods in recent years. Examples include sensing of brain intracellular calcium using conformational changes of cell permeable acetomethoxyl (AM) ester Ca^2+^ chelators mimicking optical calcium indicators but conjugated to a paramagnetic manganese atom for MRI (Barandov et al. 2019). These sensors offer specificity verified by selective response to varying doses of similarly divalent ions Ca^2+^ and Mg^2+^ modulating T1 MRI contrast solely in the presence of Ca^2+^ at single µM concentrations. Testing the sensors in rat brain showed signal increase during K^+^ infusion-induced Ca^2+^ fluxes affirming this noninvasive injectable agent for directly detecting calcium events correlated with brain activity. Other small molecule sensors can detect analytes such as gaseous nitric oxide in the brain by way of high affinity manganese-based complexes (Barandov et al. 2020). Readouts of lipopolysaccharide (LPS)-activated nitric oxide synthase and its inhibition in the brain relevant to neuroinflammatory cascades were achieved using this probe. Other successful chemically-synthesized agents can be used for inducing and visualizing neuropharmacological modulation in the brain in parallel (Bricault et al. 2020). By injecting a molecular complex comprising a Gadoteridol moiety to the GABA receptor agonist muscimol, tactile stimulation causing somatosensory cortex activation underwent spatially controlled inhibition exemplifying a molecular approach that can be broadly applied for closed-loop brain access.

As with MRI-based nanoparticle imaging agents, spatiotemporal resolution for most molecular probes sits at the 10^–4^ m × 10^0^ Hz regime compared with more rapid electronic wireless neurotechnologies. Moreover, while molecular sensors can be orders of magnitude smaller than electronic devices and even nanoparticles, they are not always capable of crossing the blood brain barrier unforced due to complexities including hydrophilicity, cellular efflux and more. Future work for all tiers of agents will require the exploitation of BBB penetration schemes, sensing from nearby microvasculature using electromagnetic, chemical, or biological strategies to maneuver wireless probes into neural tissue of interest.

## Conclusion & future prospects

In this work, we surveyed cutting-edge augmentations to wireless neural probe technologies, examined the technical challenges and ramifications of sensor size reduction and consolidation, and summarized how wireless agents for variable modalities can facilitate precision recording and stimulation of the brain and drive breakthroughs in bioelectronic medicine. To harness the exclusive capabilities of wireless micro- and nano-scale probes, their physical footprint must be aggressively minimized while maintaining recording and stimulation functionality. Because miniaturization can also lead to new forms of toxicity and adverse cellular response, excess aggregation and obstruction within the brain—complementary tools must be developed for effective sensor monitoring and evacuation. We demonstrated here how devices and probes are accommodating the increasingly integrated fields of acoustic, chemical, electronic, magnetic, and optical imaging, with emphasis on cross-modality operation for creating toolkits of agents coupled to a diverse array of recording and stimulation systems—rather than a single monolithic framework.

We expect this approach to elevate research capabilities neuroscience and pave the way for clinical deployment.

## Data Availability

Not applicable.

## Abbreviations

RF: Radio frequency
MRI: Magnetic Resonance Imaging
US: Ultrasound
fUS: Focused ultrasound
BMIs: Brain machine interfaces
ECoG: Electrocorticography
fMRI: Functional magnetic resonance imaging
TMS: Transcranial magnetic stimulation
MEG: Magnetoencephalography
fNIRS: Functional near-infrared spectroscopy
IC: Integrated circuit
CMOS: Complementary metal-oxide semiconductor
PDMS: Polydimethylsiloxane
FET: Field effect transistor
ISFET: Ion-sensitive FET
NP: Nanoparticle
BBB: Blood–brain-barrier
SPIONs: Super paramagnetic iron oxide nanoparticles
mDBS: Magnetothermal deep brain stimulation
MENPs: Magnetoelectric nanoparticles
CFO: CoFe2O4
BTO: BaTiO3
MPI: Magnetic particle imaging
MEPI: Magnetoelectric particle imaging
GVs: Gas vesicles
